# Pneumatic Quasi-Passive Actuation for Soft Assistive Lower Limbs Exoskeleton

**DOI:** 10.3389/fnbot.2020.00031

**Published:** 2020-06-30

**Authors:** Christian Di Natali, Ali Sadeghi, Alessio Mondini, Eliza Bottenberg, Bernard Hartigan, Adam De Eyto, Leonard O'Sullivan, Eduardo Rocon, Konrad Stadler, Barbara Mazzolai, Darwin G. Caldwell, Jesús Ortiz

**Affiliations:** ^1^XoLab, Department of ADVR-IIT Advanced Robotics, Istituto Italiano di Tecnologia, Genoa, Italy; ^2^Department of Biomechanical Engineering, University of Twente, Enschede, Netherlands; ^3^Department of CMBR-IIT Center for Micro-BioRobotics, Istituto Italiano di Tecnologia, Pontedera, Italy; ^4^Smart Functional Materials Research Group, Saxion University of Applied Sciences, Enschede, Netherlands; ^5^Design Factors Group, University of Limerick, Limerick, Ireland; ^6^Consejo Superior de Investigaciones Cientificas (CSIC), Madrid, Spain; ^7^Institute of Mechatronic Systems, ZHAW Zurich University of Applied Sciences, Winterthur, Switzerland

**Keywords:** soft exoskeleton, exosuit, robotic wearable device, quasi-passive actuation, legged locomotion, gait assistance

## Abstract

There is a growing international interest in developing soft wearable robotic devices to improve mobility and daily life autonomy as well as for rehabilitation purposes. Usability, comfort and acceptance of such devices will affect their uptakes in mainstream daily life. The XoSoft EU project developed a modular soft lower-limb exoskeleton to assist people with low mobility impairments. This paper presents the bio-inspired design of a soft, modular exoskeleton for lower limb assistance based on pneumatic quasi-passive actuation. The design of a modular reconfigurable prototype and its performance are presented. This actuation centers on an active mechanical element to modulate the assistance generated by a traditional passive component, in this case an elastic belt. This study assesses the feasibility of this type of assistive device by evaluating the energetic outcomes on a healthy subject during a walking task. Human-exoskeleton interaction in relation to task-based biological power assistance and kinematics variations of the gait are evaluated. The resultant assistance, in terms of overall power ratio (Λ) between the exoskeleton and the assisted joint, was 26.6% for hip actuation, 9.3% for the knee and 12.6% for the ankle. The released maximum power supplied on each articulation, was 113.6% for the hip, 93.2% for the knee, and 150.8% for the ankle.

## 1. Introduction

There is a growing interest across several fields in the use of wearable sensors and robotic technologies, including exoskeletons. To date, the most common field where such wearable devices have been applied is in rehabilitation. Examples of stationary (non-user grounded) exoskeletons include the Lokomat (Jezernik et al., 2003) and LOPES (Veneman et al., 2007). Both are used in clinical settings with the patient walking on a treadmill. Traditionally, mobile exoskeletons, or orthosis, assist paraplegic individuals when walking. They have been developed to assist with daily tasks involving movement. To restore some degree of legged motility to people with pathologies causing severe loss of mobility, rigid and bulky devices remain the conventional solution. Examples of lower limb exoskeletons are given by Farris et al. (2011) and Murray et al. (2014), where parallel robotic legs connect the users' waist and feet through kinematic chains.

When targeting assistance at people with moderate to low impairments, a further level of simplification in the device can be adopted. Examples are given by navigation assistance devices developed to address the needs of the elderly, as in Kong and Jeon (2006) and Ikehara et al. (2011), where slim designs are presented, but traditional mechanical transmission mechanisms are still employed resulting in heavy solutions.

Since the next generation of exoskeletons should address not only the degree of assistance, but also the usability/acceptance by end users, there has been an evolution from exoskeletons to exosuits. Exosuits, introduced in Awad et al. (2017), Jin et al. (2017), and Schmidt et al. (2017), have ankles, knees or hips that are supported by a soft or hybrid structure and an active tendon driven actuation system. These examples benefit from a soft wearable structure, which helps to reduce the burden of the device. On the other hand, issues (e.g., weight, power consumption, and a cumbersome design due to the inclusion batteries and other mechanical components) can arise from using active electrical motors combined with a cable or belt to actively assist joints.

The presented trend underlines the desire to avoid bulky, rigid, heavy exoskeletons, by using light, soft, and shapely wearable devices. Thus, system autonomy, usability and acceptance became the foundation of the XoSoft EU project. The XoSoft EU project consortium developed a user-centered design based, soft, modular, bio-mimetic, and quasi-passive exoskeleton to assist users with low to moderate mobility impairments, such as the elderly, and post-stroke or partial spinal cord injury subjects (Power et al., 2016; Ortiz et al., 2017).

Previous work as part of this development includes the XoSoft *Beta 1 prototype* (Di Natali et al., 2019) and a quasi-passive actuation (*QPA*) strategy for system optimization introduced in Ortiz et al. (2018), where electromagnetic clutches are used to modulate the passive elements employed to store the mechanical energy. These systems do not provide any active force, but rather transfer mechanical stored energy between gait phases. To further reduce weight and size of the proposed actuation system, soft actuators have been investigated. Soft clutches should meet the following specifications: (1) linear sliding movement to be installed along the limbs; (2) able to change load bearing capabilities under certain stimuli; (3) able to vary its initial length; (4) able to respond to the imposed motion by changing from a compliant, freely elongating condition to a stiff state.

The interest in soft actuators is evident from the literature, as demonstrated by Van Ham et al. (2009), Manti et al. (2016), and Wolf et al. (2016). In particular, soft clutches are valuable to human-robot interaction and soft robotics. The ongoing research on soft actuators aims to develop clutches, brakes, dampers and devices based either on specific phase change materials (e.g., low melting point materials, Taghavi et al., 2018, electro- and magneto-rheological fluids, Petek, 1992; Carlson and Jolly, 2000; Oh and Onoda, 2002; Nikitczuk et al., 2010; Alkan et al., 2013) or on a friction-based mechanism (e.g., electro-adhesion, Diller et al., 2016; Ramachandran et al., 2019, jamming and cable tensioning Walsh et al., 2007; Van Dijk et al., 2011). Vacuum-controlled jamming (either particles or layers) is a suitable technology that has found a number of uses within the robotics community (Brown et al., 2010; Follmer et al., 2012; Jiang et al., 2012; Stanley et al., 2013; Li et al., 2014; Zubrycki and Granosik, 2017; Sadeghi et al., 2018). In the field of wearable robotics, Hauser et al. (2017) a wearable joint support was developed based on granular jamming controlled by pressure. As highlighted in Kawamura et al. (2003), a laminated passive element could be used for rigid limb attachments (i.e., orthosis), Bureau et al. (2011) and Tonazzini et al. (2018). In wearable haptics, Mitsuda (2017) implements a force display based on fabric jamming technology, whereas layer jamming has been employed as a brake in Choi et al. (2018) and Ramachandran et al. (2019).

In Sadeghi et al. (2019a), the authors demonstrated the actuation mechanism of a textile based clutch (*TBC*) being applied to the wearable XoSoft. The *TBC* stiffness increases tens of times by applying negative pressure to two parallel textiles featuring repetitive grooves, contained within an airtight deformable chamber. The layers pack together and the grooves interconnect to restrict the relative movement. This, on a microscopic scale bears some similarity to natural muscle. The *TBC* results indicated a viable option for developing a soft controllable clutch wearable system. The braking force is determined by the friction force, which is controlled by the vacuum pressure applied between the two layers.

The major contributions of this work are the development of a new soft, wearable device, the *Gamma prototype*, that uses soft pneumatic *QPA* to deliver gait assistance. The modularity of the device is presented, and configurations for hip and knee flexion, and ankle plantar-flexion assistance are demonstrated. The system design is illustrated. The actuators are validated experimentally, followed by an assessment of the overall system to determine its effectiveness during testing on a healthy subject during the walking task. In section 2, the full design is presented. In section 3, the modeling of the exoskeleton and human interaction is addressed. In section 4 the experimental protocol and results are explained and discussed. Finally, in section 5, conclusions and future developments are addressed.

## 2. Design

The *Gamma prototype* developed within the XoSoft project, is the most advanced iteration of the user-centered design approach. The soft exosuit is tested to assess functionality, validity and reliability. The device meets the essential requirements and is classified as a class 1 medical device compliant with IEC 60601. When compared to the previous *Beta 1* variant, several issues have been addressed to improve and enhance the actual exosuit: to facilitate donning and doffing, to improve comfort and acceptability, and to test the effectiveness of use and operation with different primary users. Consequently, the *Gamma* prototype involved a truly modular approach, allowing actuation of different joints, unilaterally or bilaterally, depending on the users' needs. A variety of primary users were targeted to test wearing the *Gamma*. Thus, the current design prototype addressed all these features of modularity, and reconfigurability to subject needs, showing the potential of becoming a platform for rehabilitation or use as a daily assistive device.

**Figure 1:**
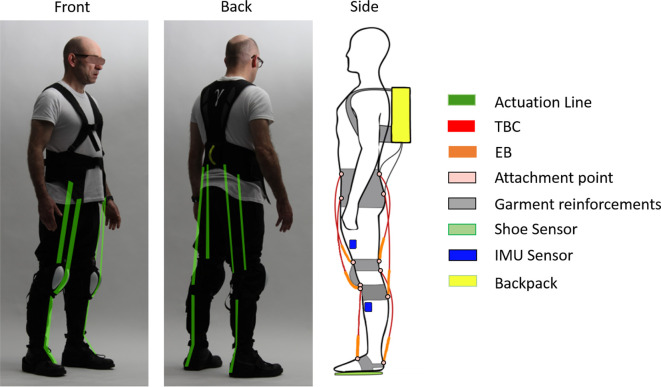
Overview of the technologies integrated in the *prototype Gamma*. The backpack contains: the pneumatic system, electronic boards and the central computer. The front and back views show in green, all the possible QPAs lines that can be installed on the garment. The side view displays for each actuator line the *TBC*s and related EBs, attachment points, shoe sensors, IMU sensors placement, garment reinforcement points, and backpack. Written informed consent was obtained from the individual pictured in the figure.

**Figure 2:**
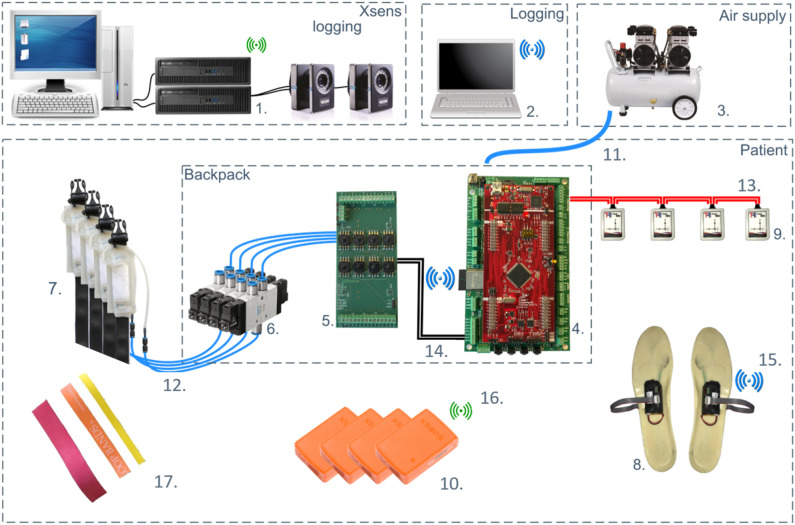
Overview of the system components and layout for *Gamma prototype* version. (1) Xsens logging; (2) Exoskeleton state and sensors logging; (3) Air compressor; (4) Central processor; (5) Valve controller board; (6) Valves; (7) TBCs; (8) Shoe sensors; (9) IMUs (for control); (10) Xsens system (for motion tracking); (11) Compressed air; (12) Vacuum lines; (13) CAN bus 1; (14) CAN bus 2; (15) Wi-Fi network 1, (16) Wi-Fi network 2; (17) EBs. Main hardware components of the exoskeleton are described in section 2.1.

Figure 1 shows pictures and a technical drawing of the actuator arrangement on the soft exoskeleton including: sensing/sensors, the QPA technologies, and the garment centered mechanical structure of the *Gamma prototype*. The main technical elements composing this rehabilitation platform are presented in Figure 2, and are summarized as follows:

- **TBC**: A mechanical clutch actuated by vacuum pressure to block the mechanism that allows the controlled storage and release of energy.
- **Elastic Band (EB)**: Elastic element to store mechanical energy. On one side (distal attachment) it is connected directly to the body segment. The other side (proximal) is connected to one end of the *TBC*.
- **Body Attachment**: Body attachment required to transmit the forces from the actuators to the wearer's body.
- **Shoe Sensors**: Sensors placed on the plantar side of the foot to detect the contact between the foot and the floor. Used as an input for the motion/gait segmentation.
- **IMU Sensors**: Sensors placed on each thigh and shin to detect the knee angular displacement. Used as an input for the motion/gait segmentation.

The actuation principle is defined as Quasi-Passive Actuation and it is composed of an EB and the *TBC* in series. *QPA* refers to any controllable element that cannot apply a non-conservative motive force (Endo et al., 2006). Thus, *QPA* includes any combination of variable-dampers or clutches in conjunction with passive components such as springs. The work presented in Di Natali et al. (2019) on the previous soft wearable device (*Beta 1 prototype*), introduced the *QPA* unit for a unilateral hip and knee assistive configuration. The modular design description and assessment is also addressed. In particular, the *Beta 1 prototype* was designed and tailored for a unique patient with a lower limb mobility problem on the right side. unlike the *Beta 1 prototype*, the control system of the *Gamma* uses a biomimetic approach based on gait segmentation to appropriately activate or deactivate the *QPA* to alternate between the storing and releasing actuation phases. The control exploits insole sensors and IMUs feedbacks to identify gait patterns.

### 2.1. Design Description

#### 2.1.1. Garment

The *Gamma prototype* garment was developed using feedback from the tests with the *Beta 1 prototype*. To define a fabric suitable for the prototype, the following properties are taken into account: fiber composition, weight, elongation/stretch, absorbency/wetting and comfort (Mecheels and Umbach, 1977). A one-size-fits-all garment was developed for all the participants by ensuring optimal fitting for the key measurements. To improve comfort and breathability, soft fabric (100% polyester 75D Interlock fabric, 210 grams per yard) was selected for the main pants section of the garment.

Reinforced elements such as Polyester webbings and Nylon 210D have been used to enable sufficient inelastic response on the body attachments where forces will be applied. The main webbings and reinforcements are displayed in gray in Figure 1. To avoid any contact between the front knee actuation and the patella bone, a protective plastic cup is employed to rise the actuation from the knee profile (displayed on the lateral view of Figure 1). Loose and elastic fabric are used to facilitate quick donning. The overall weight of the garment is 0.8 kg.

#### 2.1.2. Actuation Units

As previously mentioned, the actuation is defined as quasi-passive since the system stores energy from the user during certain gait phases delivering it when needed. The actuation unit is composed of a EB and pneumatic clutch (*TBC*) in series. The EB and *TBC* assembly is able to modulate the assistance to the user, by controlling the storing and releasing phases of the mechanical power. If the clutch is not engaged, the actuation line results are neutral to the user, as no storing or releasing phase is involved, while the user is moving. The *TBC* is an assembly of two inextensible webbings equipped with an array of 2.3 mm wide parallel rigid bumps (similar to a rack gear) fabricated directly on them by a hot embossing technique, and two elastic bands. Internally, within the same envelop, the recall elastic element is connected to both parallel layers. These elastic bands have an intrinsic elasticity which creates a small constant stiffness. This ensure that the TBC-EB unit is always under small tension during movement, remaining tight against the limbs. The complete unit is air sealed by a silicone elastomer cover (Sadeghi et al., 2019b). When the vacuum pressure is applied, the *TBC* system is engaged, with the *TBC* stiffness characteristic changing from elastic to rigid along the pulling direction. When the clutch is engaged, it becomes stiff and the elastic band starts elongating as the user is moving the joint.

From an implementation standpoint, and based on previous studies and simulations by Ortiz et al. (2018) and Di Natali et al. (2019), it is possible to characterize each specific actuation line for the hip, knee and ankle joints by maximum length, elongation, relative angular displacement and predicted force provided by the actuation. When the clutch is disengaged, unappreciated force is exerted by the system to the user and this configuration is defined transparent.

**Figure 3:**
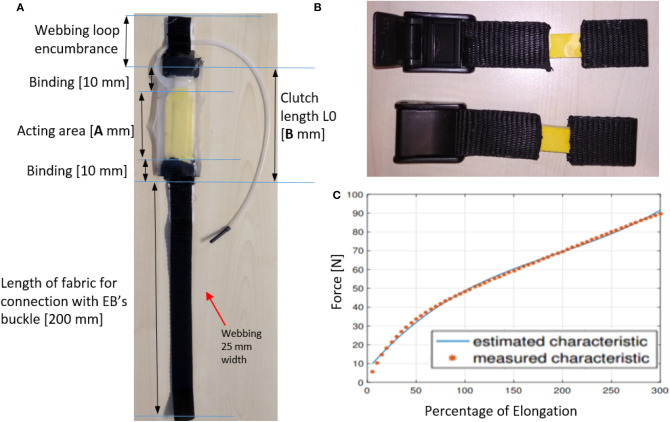
**(A)** *TBC* sample. **(B)** EB sample. **(C)** EB stiffness characteristic and its third order interpolation.

The initial length and maximum elongation associated with each actuation segment are measured on an average size subject as defined by Dempster and Gaughran (1967). These data are provided in Table 1 and the *TBC* geometry is displayed in Figure 3A. In Table 1, “max travel” represents the maximum elongation permitted by the *TBC* if actuation is not needed and the system has to be transparent to the user. Based on the information in Table 1, two families of clutches have been developed. The first family is used for the upper leg (hip and knee flexion-extension) and the second family for both ankle plantarflexion and dorsiflexion. Table 2 provides the *TBC* characteristics. Each clutch has a webbing loop on one side for quick installation as shown in Figure 3A. The webbing loops on both sides of the actuation line connect it to the garment using adjustable dog-bone anchors. The *TBC* terminates with approximately 200 mm long webbing that connect to a cam buckle, and then to the EB, (Figure 3B). This set-up permits adjustments of the distance between *TBC* and corresponding EB.

**Table 1:** Geometrical characteristics collected to guide the *TBC* and EB design.

**Joint**	**Max force [N]**	**Actuation line length**	**TBC travel (max travel) [mm]**	**Expected elastic elongation [mm]**
Hip extension	30	200–250	50 (100)	50
Hip flexion	30	300–400	50 (100)	50
Knee extension	30	250–350	50 (100)	50
Knee flexion	30	200–300	50 (100)	50
Ankle dorsiflexion	20	220–280	20 (40)	20
Ankle plantarflexion	60	250–300	20 (50)	30

**Table 2:** *TBC*s design characteristics.

**TBC family**	***TBC L*_0_ (B) [mm]**	**Length of interlocking zone (A)**	**Requested resistance force [N]**	**TBC travel (max travel) [mm]**	**Pressure [bar]**
Upper leg	140	110	100	50 (100)	0.3
Lower leg	110	80	200	20 (50)	0.3

*Length of interlocking zone (A) and initial length (B) are related with the figure displayed in Figure 3A*.

The EBs used in the *Gamma prototype* are latex-made elastic bands connected to the webbing material through a vulcanization process. These are displayed in Figure 3B. Table 3 shows the EB's design characteristics, based on the assistance required. The EBs stiffness characteristics are reported in Table 4, as a third order function of elongation percentage as in Equation (1) (the elasticity trend is displayed in Figure 3C)

(1) $$\begin{array}{l} f_{k}\left(\alpha_{k}\right)=a_{3}\Delta L_{k}^{3}+a_{2}\Delta L_{k}^{2}+a_{1}\Delta L_{k} \end{array}$$

where Δ*L*_*k*_ is the percentage of elongation.

**Table 3:** EBs design characteristics.

**EB family**	***EB L*_0_ [mm]**	**System length [mm]**	**Nominal Stiffness [N/%]**	**EB typology**
Hip extension	20	100	0.5	A
Hip flexion	50	150	0.8	B
Knee extension	30	150	1	C
Knee flexion	25	120	0.5	A
Ankle dorsiflexion	10	100	0.25	D
Ankle plantarflexion	20	140	10.5	A

**Table 4:** EBs typology.

**EB typology**	**Nominal stiffness [N/%]**	***a*_3_**	***a*_2_**	***a*_1_**
A	0.5	3.66*10^−6^	−0.002	0.565
B	0.8	9.06*10^−6^	−0.005	1.194
C	1	1.23*10^−5^	−0.007	1.621
D	0.25	2.04*10^−6^	−0.001	0.312

*EB's stiffness as a third order polynomial fitting coefficient as in Equation (1)*.

The garment has webbing anchor straps (shown in Figure 1) providing the body attachments for proximal and distal ends of the actuation lines. The waistcoat provides the body attachments for frontal and rear knee and hip actuations. At the knees, two webbings loops for body attachment are positioned, one below and one above the joint. For the distal attachment of ankle actuation, straps are worn over regular shoes providing attachment points on the front and back of the foot. The *TBC*s are connected to the garment through webbing-loops and dog-bones.

#### 2.1.3. Pneumatic System

**Figure 4:**
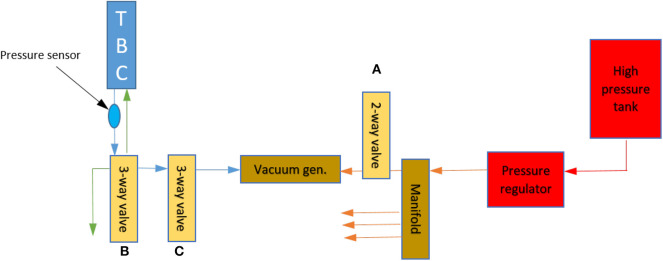
Pneumatic circuit for *TBC* control. The system pressure is represented with a color map. The green arrow is for pressure of 101*kPa*, blue is pressures up to 10*kPa* and below 101*kPa*. The orange represents an intermediate pressure up to 6 bar (600*kPa*), and the red represents the high line pressure of between 600 and 1, 200*kPa*.

The *TBC* is activated pneumatically by applying negative pressure. *TBC*s are managed by means of pressure sensors and solenoid valves to connect the *TBC* to the vacuum line, atmospheric pressure and a closed state. The closed state is provided to allow the *TBC* to maintain the set pressure (idle state). The primary source of the pneumatic system is a compressor or air line (if present in the clinic). The pneumatic control system schematic for the exoskeleton is shown in Figure 4. The pressure regulator sets the low pressure supply (less than 6 bar) to the exoskeleton's pneumatic line. A 2-way valve (MHJ10-S-2,5-QS-6-HF, electro-valve, Festo Inc., Germany), represented by A in Figure 4, is connected in line with a vacuum generator to provide a vacuum when needed. The vacuum generator (VN-20-H-T6-PQ4-VQ5-RO2, vacuum generator, Festo Inc., Germany), which uses the Venturi principle, can generate vacuum pressures up to −90 kPa with an air flow of 100 L/min. A double silencer ensures that the noise is under 50 dB. The vacuum generator is connected to the *TBC* through two series connected of 3-way solenoid valves (MHE2-MS1H-3/2G-QS-4-K and MHE2-MS1H-3/2O-QS-4-K, electro-valve, Festo Inc., Germany). The first valve, in Figure 4, represented by B activates if the clutch needs to be engaged. The second 3-way valve (represented by C) connects the *TBC* to the external environmental pressure. If both the 3-way valves are off, the *TBC* is isolated and the internal pressure is maintained. The opening and closing of valves is controlled by a pressure sensor (MPXV6115V, Absolute Pressure Sensor, Freescale Semiconductor, pressure range −115 to 0 kPa). To control multiple actuators in parallel, a multi-way manifold is placed between the pressure regulator and the vacuum generator. The Valve Driver Board is a custom design based on a Texas Instruments microcontroller (TMS320F28035) with a CAN transceiver to receive commands and return status, log and pressure sensor values.

#### 2.1.4. Sensors

A pair of custom made, soft foam (Hekapur PU, Exact Plastics, Bröckel, Germany) insoles with four embedded force sensing resistors (FSR, 1-Inch ShuntMode, Sensitronics, Bow, USA) are used to detect foot contact with the ground. There is one sensor under the heel, two under the metatarsal area and one under the phalanges (Figure 2). The pressure sensors, operating as switches provide a digital signal depending on whether a pressure threshold is exceeded. As shown in Figure 1, four IMUs (Tech-MCS V4, Techaid S.L., Madrid, Spain) are placed at the shin and thigh of each leg. The signal information is processed to extract the knee joint angular displacement (Cooper et al., 2009). The method directly uses the orientation matrices provided by these sensors. By fusing the sensor outputs from the ground contacts and knee angles [heel pressure signal (HS), outside insole pressure signal (OS), inside insole pressure signal (IS), toe pressure signal (TS), and the derivative of the knee angular displacement (KAD)], in real-time, the system segments the gait cycle to identify different events such as: *heel strike, flat foot, front foot, toe off* , *positive speed inflection* and *negative speed inflection*. Using this sensory data and sequencing, the specific control signals can be applied to provide the required assistance and actuator action (**Figure 6**).

#### 2.1.5. Electronics and Communication

The electronics consists of two Wi-Fi modules (ESP32, Espressif, Shangai, China), for insole sensor readings, transmitting to the main central processing unit (CPU) board (Launchpad XL Development Kit, Texas Instruments, Dallas, US) with a custom extension cape board providing Power Management, I/Os and CAN Communication as shown in Figure 2. The IMU sensors communicate with the CPU through CAN bus communication. The CPU has all the peripherals needed to run all the communication; the firmware has been developed to be able to link with the Control Valve Board. The Power Supply Board and Valve Driver Board act as a decoupling interface to separate the low power signals used by the CPU from the high power signals to the valves and for CAN communication. The form factor of this board allows boards to be stacked below (Power Supply) and above (Valve Driver), to create a modular and easily replaceable system. The Power Supply board is a custom board providing enhanced I/O and communication capabilities. It has CAN transceivers and Wi-Fi modules. Each ESP32 Wi-Fi module sends the FSR data wirelessly to the CPU. The board provides control signals and power to all the pneumatic valves (both 2-way and 3-way) using a power MOSFET and a freewheeling diode. The microcontroller draws energy from the Texas Instruments LaunchPad XL board. A 3-way connector links the pin of the CAN transceiver available on the LaunchPad, providing a unique way to assemble the two boards together. The pressure sensors are sensitive to negative pressure only, maximum −15 kPa. Each sensor can be easily plugged into the corresponding vacuum line for monitoring.

The electronics and pneumatic system, together with an embedded battery (25.2 V nominal voltage and 4 Ah capacity), are contained in the backpack which has a total weight of 4 Kg.

#### 2.1.6. Control Strategy

**Figure 5:**
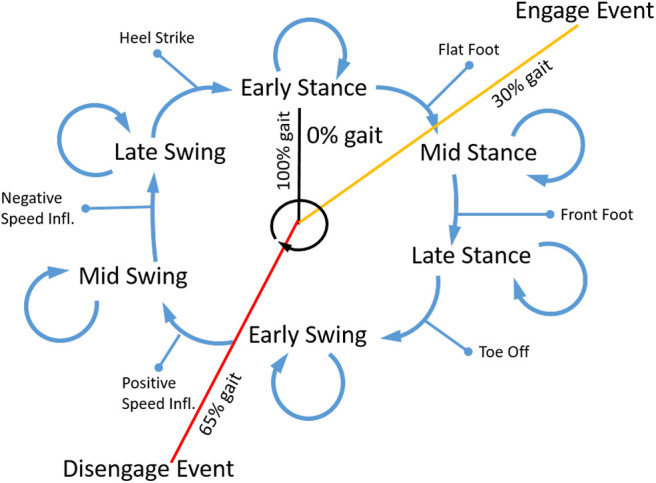
Control flow of the FSM, in which the events (*heel strike, flat foot, front foot, toe off* , *positive speed inflection* and *negative speed inflection*) determining the state changes are reported.

**Figure 6:**
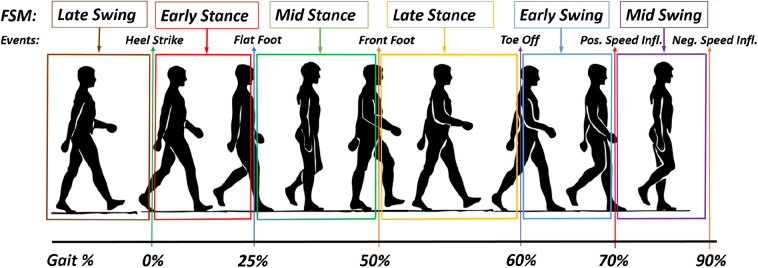
Schematic of the gait event and FSM.

Actuation of the *XoSoft* *QPAs* is linked with the motion of the user. Due to the geometry of the exosuit the torque delivered by the actuation system is dependent on the moment arm *R*_*k*_(α_*k*_) subtended by the cord *L*_*k*_, and by the cord elongation Δ*L*_*k*_, as described in section 3. This is shown in **Figure 8** where the cord corresponds to the actuation line, which is the series connection of the *TBC* and EB from the proximal to the distal body attachments. The system stores and delivers energy as a consequence of the joint rotation. During the first phase of energy storage, a resistive torque is generated, which the user has to fight against. Once the joint starts rotating in the opposite direction (phase two), the system releases this accumulated mechanical energy. During the second phase, the system provides assistance to the user until the leg segment returns to the initial position (it corresponds to joint angle when the actuation has been engaged at the beginning of phase one). Therefore, it is possible to store and deliver different amounts of energy by modulating the timing of clutch engagement. The main control algorithm provides the segmentation of the gait cycle, which is shown in Figures 5, 6 as a logic state of a Finite State Machine (FSM). In correspondence of each state, the clutches can be set or reset.

The power delivered by the EB is controlled by detecting the phase in the gait cycle and using this information to engage and disengage the clutches. Figure 5 shows the flow chart of the state machine used to regulate these activations. The system tracks the gait phases sequence, using the foot contact data and knee angle, to determine when to engage, disengage or keep the clutch current state. The inputs to the stateflow are the foot contact signal and knee angle. The outputs of the stateflow are the on-off clutch activation signals. A configuration file is used to set and save each subjects' profile of who uses and tests the exosuit. It reports and specifies the actuation configuration such as the leg (left/right/bilateral), the joint and movement to be assisted (i.e., hip flexion and extension, knee flexion and extension, and ankle plantarflexion and dorsiflexion). The control of each clutch is set by means of the activation and deactivation instant during each percentage of gait. Therefore, the leg logic blocks detect the gait events sending the appropriate signals to the activation blocks, where the corresponding clutch control signals are triggered. Thus, the device is controlled with an open-loop algorithm. Apart from each of the clutch states, on the high level of the control, there is not a physical quantity that has to be controlled.

In the *Beta 1 prototype*, knee angle detection was unavailable. A simpler segmentation approach was used, and it relied on the time measurements between consecutive toe off (TO) and heel strike (HS). Thus, the swing phase estimation was based on the timing of the previous step. This simple approach is limited to scenarios where smooth changes in cadence are assumed. Therefore, to improve the gait segmentation, this method based on knee angle detection has been developed.

Based on the data from the shoe insole sensors and the IMUs, the controller is able to determine the six gait phases that form the finite state machine (FSM) (*early stance, mid stance, late stance, early swing, mid swing*, and *late swing*). These are shown in Figure 6. The assistance provided to the user, which is based on the user needs, is designed accordingly to the actuation configuration and the actuation strategy (clutch engagement and disengagement events expressed as gait percentage). The control designer, (essentially the therapist) may select the point, expressed as a percentage, of gait when the actuation should engage and disengage. The control system then processes this information to determine the correct timing to switch the actuator state. For every subsequent gait cycle, each duration os each segment is regenerated based on the previous gait's total time.

**Figure 7:**
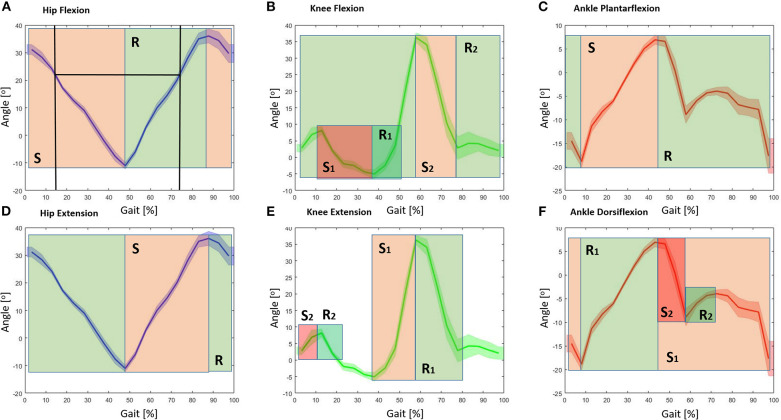
Schematic of possible control strategies for each actuator group (hip, knee, and ankle) as a function of the joint angle. The green areas represent the energy releasing phase (R), whereas the red areas represent the storing phase (S) for each actuator grouping. **(A)** Representation of the hip flexion, the joint motion/rotation is associated with where energy could be stored and released. The black vertical lines show the instant of engagement and disengagement of the *TBC*, where the clutch is engaged at the 15% point of the gait cycle, and maximum releasing phase at 75%. **(B)** Storing and releasing phases associated with the knee flexion. **(C)** Storing and releasing phases associated with the ankle plantarflexion. **(D)** Storing and releasing phases associated with the hip extension. **(E)** Storing and releasing ranges associated with the knee extension. **(F)** Storing and releasing phases associated with the ankle dorsiflexion.

Figure 7 gives an example of assistance, separating the storing and releasing phases, for each assisted joint. Because of the design of the *QPA*, the actuator needs to store energy to be able to provide assistance. In Figure 7A, an example of hip flexion actuation is presented. The first vertical line represents the instant of *TBC* engagement (at about 15% of the gait cycle). Naturally, the storing phase ends with the minimum angle reached by the joint (at about 50%), then the releasing phase starts. The releasing phase will terminate as soon as the initial joint angle (instant of engagement) has been reached or as soon as the *TBC* is disengaged by the controller. In this example, the releasing phase ends at approx. the 75% of the gait cycle. Figures 7B,E represent the knee movement (flexion and extension respectively) and show the ranges over which energy is stored and released. Since the knee angle rotation plot has two local maximums, two storing phases (*S*_1_ and *S*_2_) and releasing phases (*R*_1_ and *R*_2_) are possible. Of course, this will result in a different sensation for the user, and different assistance profiles (Di Natali et al., 2019 assesses the knee flexion control strategy). Figure 7F shows the two possible actuation strategies for ankle dorsiflexion. Thanks to the two local maximums, the ankle dorsiflexion can be assisted during the whole gait cycle (*R*_1_ and *S*_1_) or (*R*_2_ and *S*_2_) around the toe-off event (60% of gait cycle). The control strategy *S*_2_-*R*_2_ can be used to reduce the foot drop issue.

## 3. Modeling

**Figure 8:**
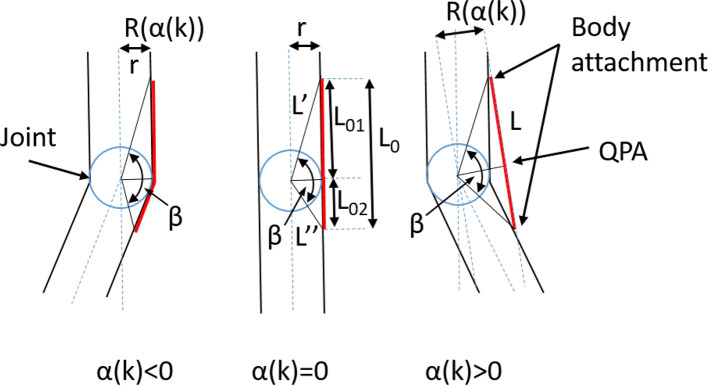
Geometrical representation of the actuation unit.

A wearable exosuit/exoskeleton is designed to aid the user by applying a percentage of the needed power to accomplish the task. In this instance, the exoskeleton is applying force on the user's leg segments. Each *QPA* provides pulling forces between the proximal and distal body attachments. The body attachments are below and above the assisted joint, as shown in Figure 8. The force generated by the EB is a function of its elongation. The interaction with the user is important since joint motion will drive the EB by providing elongation. Figure 8 summarizes the main mathematical elements related to the force/torque transmission. The cord length (the in-line combination of the *TBC* and EB) *L*_*k*_(α_*k*_), is a function of the joint angle α_*k*_, where the index _*k*_ could be the front or rear hip, knee and ankle joint. The user's joint rotation generates an elongation defined as Δ*L*_*k*_ on the cord. The total cord length is governed by Equation (2).

(2) $$\begin{array}{l} L_{k}\left(\alpha_{k}\right)=\left\{ \begin{array}{cc} \sqrt{L_{k}^{\prime 2}+L_{k}^{\prime \prime 2}-2L_{k}^{\prime }L_{k}^{\prime \prime }\cos\left(\beta_{k}\right)} & \text{if }\alpha_{k}\text{is}\geq 0\\ L_{0k}+r_{k}\alpha_{k} & \text{if }\alpha_{k}\text{is}<0 \end{array}\right. \end{array}$$

where

(3) $$\begin{array}{l} L_{k}^{\prime }=\sqrt{L_{01k}^{2}+r_{k}^{2}} \end{array}$$

(4) $$\begin{array}{l} L_{k}^{\prime \prime }=\sqrt{L_{02k}^{2}+r_{k}^{2}} \end{array}$$

The angle β_*k*_ is equal to:

(5) $$\begin{array}{l} \beta_{k}=\pi -\alpha_{k}-\arctan\left(\frac{r_{k}}{L_{01k}}\right)-\arctan\left(\frac{r_{k}}{L_{02k}}\right) \end{array}$$

*L*_0*k*_ is the length of the cord formed by the *TBC* and EB assembly in its neutral position. This occurs when α_*k*_ = 0, it is equal to:

(6) $$\begin{array}{l} L_{0k}=L_{k}^{\prime }+L_{k}^{\prime \prime }=L_{01k}+L_{02k} \end{array}$$

The torque provided by the actuation system is strongly influenced by the moment arm *R*_*k*_(θ_*k*_) subtended by the cord *L*_*k*_, and by the cord elongation Δ*L*_*k*_. The cord elongation is completely provided by the EB's elongation, whereas the *R*_*k*_(α_*k*_), function of the joint angle is expressed as:

(7) $$\begin{array}{l} R_{k}\left(\alpha_{k}\right)=\left\{ \begin{array}{cc} r_{k} & \text{if }\alpha_{k}\text{is}<0\\ \frac{L_{k}^{\prime }L_{k}^{\prime \prime }}{L_{k}\left(\alpha_{k}\right)}sin\left(\beta_{k}\right) & \text{if }\alpha_{k}\text{is}\geq 0 \end{array}\right. \end{array}$$

As a first approximation, *R*_*k*_(α_*k*_) is considered to be always aligned to the sagittal plane. Therefore, the torque generated on the assisted joint is proportional to the EB's force, *f*_*k*_ as shown in Equation (8).

(8) $$\begin{array}{l} \tau_{k}=R_{k}\left(\alpha_{k}\right)f_{k}=R_{k}\left(\alpha_{k}\right)K_{EB}\left(\Delta L_{k}^{EB}\right) \end{array}$$

Where $K_{EB}\left(\Delta L_{k}^{EB}\right)$ is the third order EB's stiffness function reported in Table 4 and $\Delta L_{k}^{EB}$ is the amount of elongation generated by the EB.

Tasks conducted by the test subject while wearing the soft exoskeleton are characterized by a particular amount of torque τ_*t*_ at each joint. The exoskeleton aids the user by providing the torque τ_*ex*_, as defined in Equation (8). The mechanical system, comprising both the user and exoskeleton, requires an amount of energy for the storing phase. This energy is provided by the user during a specific phase of the gait cycle. The total absorbed energy, into the EB, is then returned by the exoskeleton in the form of assistance.

To analyze the system's behavior, the power associated with the task, which is the combination of the torque and angular velocity of each joint, is calculated in Equation (9).

(9) $$\begin{array}{l} P_{t}=\tau_{t}\overset{\cdot }{\alpha_{t}} \end{array}$$

It is then possible to calculate the assistance provided by the exoskeleton by computing the ratio of powers (Λ) as:

(10) $$\begin{array}{l} \Lambda =|\frac{P_{t}-P_{ex}}{P_{t}}-1| \end{array}$$

where *P*_*t*_ is the measured power of the user when not wearing the exoskeleton, and *P*_*ex*_ is the power generated by the exoskeleton. Λ = 0 means that the exosuit is not providing any assistance, while Λ = 1 represents 100% of assistance (the exoskeleton assistive power is instantaneously equal to the user power).

## 4. Experimental Evaluation

**Figure 9:**
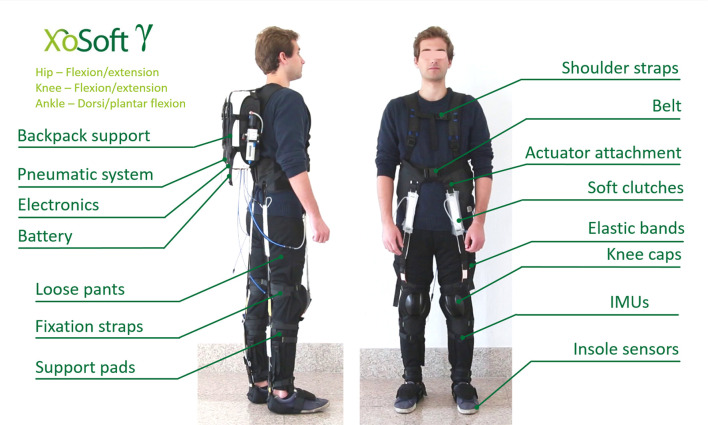
The system overview of the *Gamma* prototype used during the experimental evaluation. Written informed consent was obtained from the individual pictured in figure.

This section characterizes the *TBC* and pneumatic system. This is followed by an assessment on a healthy subject (the subject had a traumatic event in 2012 on its right ankle, causing compensatory movements) to evaluate, from a control system perspective, the assistance provided (Figure 9). The test evaluation is compliant with the experimental protocol accepted by the Ethical Committee of Liguria, Italy (protocol reference number: CER Liguria 001/2019) and the subject gave written informed consent in accordance with international guidelines. The software was evaluated during a 10 min walking task on a treadmill, during which the exoskeleton's parameters, together with motion tracking, were recorded. A second test was conducted along a 10 m straight path where the kinematics of the limbs and ground reaction forces were measured to analyze the effectiveness of the transmitted assistance.

### 4.1. Actuator Characterization

**Figure 10:**
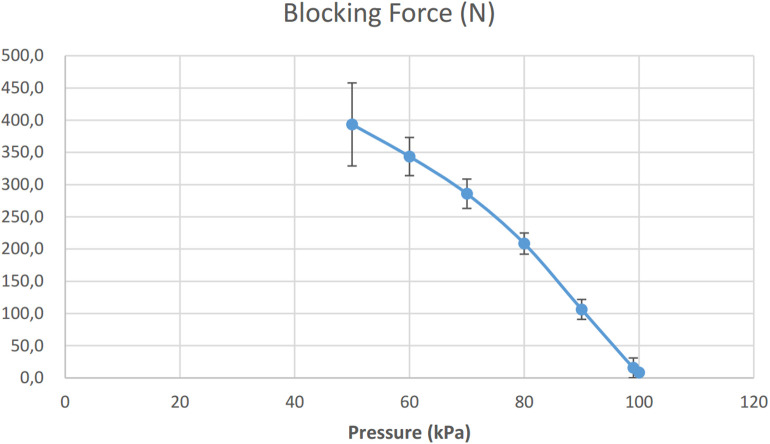
The average maximum force of *TBC* recorded at different applied pressures (for easy conversion 1atm = 101.3 kPa, 50 kPa corresponds to 50% of vacuum).

The actuators have been evaluated mechanically, pneumatically and electrically to characterize the actuation timing, the generation of forces and the power consumption. Each actuator is composed of an *EB* and the *TBC*. Each *TBC* is supplied by a pneumatic system, which is controlled by electromagnetic valves. The *EB* characterization has previously been evaluated in Di Natali et al. (2019). As previously mentioned, the stiffness characteristic of these latex *EBs* is not linear. A 3rd order polynomial has been used to fit the elastic behavior as a function of the percentage elongation (Δ*L*_*k*_), as in Equation 1. The *TBC* blocking force has been evaluated by measuring the resistive force generated as the vacuum pressure is increased. An Instron (ITW, Glenview, IL) force measurement system was used to characterize the *TBC* (Figure 10). The tests were repeated ten times for the lower leg clutch (the smaller clutch of the *TBC* family, Table 2) with 100% overlap between the two layers of the device. The tests were performed starting with a null vacuum pressure (101*kPa*) and reducing (increasing vacuum) in steps of 10*kPa*, to 50*kPa*. The profile although not linear, shows a smooth repeatable response increasing to a maximum blocking force of 400*N* at a pressure of 50*kPa*. A maximum blocking force of 540*N* was recorded at a pressure of about 42*kPa*, however at this point the *TBC* broke. Therefore for safe operation a peak pressure of 50*kPa* was selected, which gave a peak blocking force of 400*N*. In absence of vacuum, the average recall force is 7.5*N*. This is due the intrinsic recall elastic element into the *TBC*.

As each actuator forms a load for the vacuum system it is believed that increasing the number of actuators could impact the operational timings of the exoskeleton. To test this the activations timing for single actuator and four actuators were tested. In these test the single actuation and the four actuator units were connected to the vacuum system. To determine the time needed to actuate these clutches, ten tests were performed for each of two families of clutches. The times to create a vacuum pressure of 70*kPa* were measured. The time to actuate the upper leg clutches, is 0.10±0.006*s* for the single clutch and 0.18±0.026*s*, if four clutches are connected to the vacuum generator. For the lower leg clutches, the time of actuation is 0.08±0.018*s* for the single clutch, and 0.12±0.010*s*, if four clutches are connected to the vacuum generator. The releasing phase was also measured. The time, always constant, was 0.1*s* no matter of how many clutches were controlled at the same time. Therefore, we could assume an average time to energize and released each actuation of 0.12*s*. The test shows a dependency between the time of actuation and the number of clutches connected to the same vacuum generator. This is probably because the characteristics of the Venturi method used to generate vacuum and the selected pneumatic circuitry (shown in Figure 4). As previously mention in section 2.1.3, the Venturi system employs high air flow to suck air generating vacuum. Thus, the characteristic latency measured to energize the clutches is due to the air contained into the clutch and along the pneumatic lines of the exoskeleton.

To evaluate exoskeleton autonomy, the *Gamma prototype* was tested on a treadmill for approximately 10 min at a constant speed of 3 km/h. To produce comparable results with the system assessed in the section 4.3 the device was configured with six actuators (two actuators for hip flexion, two actuators for knee flexion, and two for ankle plantarflexion). During the walking task, the actuator engagement sequence, accordingly to the designed control strategy (more details are in section 4.3), was driving the actuation of each clutch at a frequency of 0.85 Hz. During testing, the overall current consumption (including CPU, sensors, communication and actuations) was monitored and measured. The power consumption, in terms of average current, is 0.17 A for each actuator. Regarding autonomy, the *Gamma prototype* equipped with six actuators assisting walking at 3 km/h last approximately 4 h (235.3 min).

**Figure 11:**
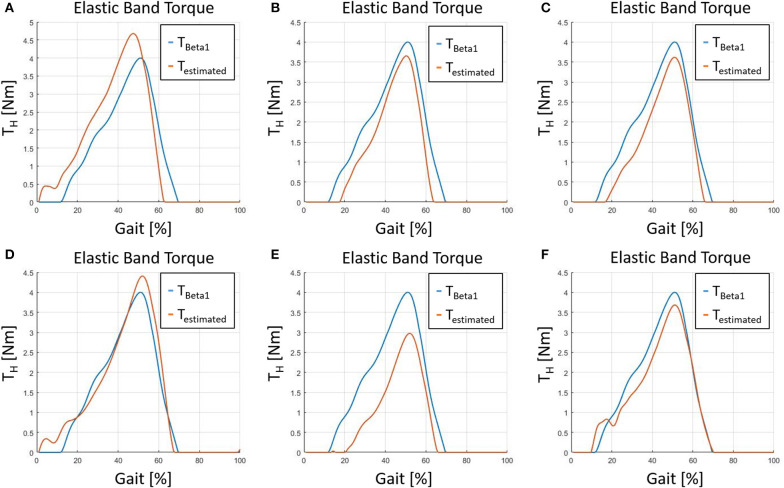
The figure shows six different tests where the torque was measured during the assessment of the *Beta*1 prototype in Di Natali et al. (2019) against the torque estimated with the geometrical model presented in section 3. **(A)** Test 1, **(B)** test 2, **(C)** test 3, **(D)** test 4, **(E)** test 5, and **(F)** test 6.

To assess the efficacy of the exoskeleton, the forces generated by the EBs during operation should be measured. A possible method would consist on measuring the EB's elongation with optical methods. In Di Natali et al. (2019) such data was gathered during the system assessment of the *Beta*1 prototype. During the tests, different control strategies were evaluated and the EB elongations measured using optical system in combination with two in-built markers. Two optical markers were installed at both ends of each EB to directly measure their elongation during operation. Since the optical system was not available for the current system evaluation, the geometrical model presented in section 3 is validated on the data gathered during the *Beta*1 prototype assessment presented in Di Natali et al. (2019) (employing the measurements of the EB's elongation). The torque generated by the EB elongations were estimated with the model presented in section 3. Figure 11 reports the results of the measured torque of the *Beta*1 prototype against the estimated torque employing the geometrical model. The mean absolute error and standard deviation are 0.25±0.31*Nm*, whereas the mean relative error with respect to direct measurement and its standard deviation are 6.52±8.13%. Thanks to the validation of the geometrical model, and considering an error below 7%, the model can be considered as having a good level of reliability. The geometrical model is used to estimate the torque generated by the exosuit for the calculation of its performance in section 4.3.

### 4.2. Control Algorithm Characterization

**Figure 12:**
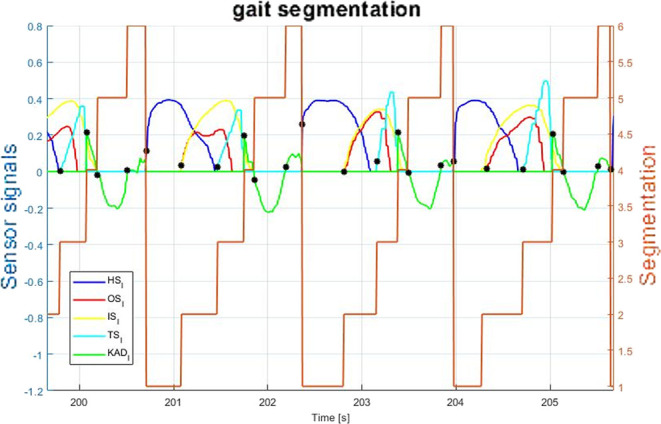
Segmentation based on sensors fusion. The signals are as follows: (HS) heel pressure signal, (OS) outside insole pressure signal, (IS) inside insole pressure signal, (TS) toe pressure signal and (KAD) the derivative of the knee angle.

The control algorithm was assessed by walking on a treadmill for 10 min at a constant speed of 3*km*/*h*. Sensor readings of the exoskeleton were recorded. To record full body kinematic and ground contact data, motion tracking was carried out using a Xsens wearable motion tracking system (MTw Awinda 3D Wireless Motion Tracker, Xsens Technologies B.V. Enschede, The Netherlands). As previously mentioned, the control algorithm is based on sensor fusion of data from two insole pressure sensors and four IMUs on the thighs and shins. This set-up identifies, for the right and left sides, six events: (i) heel strike, (ii) flat foot, (iii) front foot, (iv) toe off, (v) maximum positive speed, and (vi) minimum negative speed of the knee during swing. Figure 12 shows the segmentation achieved by applying this sensor fusion based algorithm. Thus, the gait is divided in six segments, three during stance and three during swing as follows: early stance (ESt), mid stance (MSt), late stance (LSt), early swing (ESw), mid swing (MSw), and late swing (LSw). For each segment the mean duration and standard deviation are reported in Tables 5, 6 for right and left side, respectively. The tables show repetitive and reproducible values of time lengths of each segment with a mean standard deviation over mean segment of about 19%. Each of the six segments were determined, for each gait cycle during the 10 min test, without generating any segmentation error, thus, corresponding to a 100% of execution. These events (gait percentage) are used to identify the consecutive instants of the segmentation algorithm. Differences in segment duration between the right and left side (see Tables 5, 6) are due to walking pattern asymmetry. The user reported a traumatic event on the right ankle that caused such pattern asymmetry.

**Table 5:** Right side segmentation timing and gait percentage.

**Segment**	**Mean segment duration [s]**	**STD segment duration [s]**	**Mean duration percentage [%]**	**Gait percentage [%]**
ESt	0.4279	0.1225	27.47	27.47
MSt	0.3647	0.1036	23.42	50.89
LSt	0.0855	0.0516	5.49	56.38
ESw	0.1961	0.0437	12.59	68.97
MSw	0.3413	0.0396	21.91	90.88
LSw	0.1420	0.0790	9.12	100.00

**Table 6:** Left side segmentation timing and gait percentage.

**Segment**	**Mean segment duration [s]**	**STD segment duration [s]**	**Mean duration percentage [%]**	**Gait percentage [%]**
ESt	0.3336	0.1158	21.50	21.50
MSt	0.3868	0.1127	24.93	46.43
LSt	0.2185	0.0891	14.08	60.51
ESw	0.1187	0.0365	7.65	68.16
MSw	0.3296	0.0348	21.25	89.41
LSw	0.1643	0.0503	10.59	100.00

In fact, the asymmetric gait pattern has been properly detected by the actual segmentation together with the suggested wearable sensors, confirming the validity of the proposed method. From a control point of view, these events represent the gait phases in which the *QPA* may store or release energy as demonstrated in Figure 7. Also, it is important to underline that the storing phase may occur only if the *EB* is elongated. Thus, by considering minimum and maximum angular displacement related to the particular joint motion, the control may be set accordingly to trigger the *QPA* within the desired gait areas. The next section details a particular control scenario to evaluate the assistance of the *QPA* on the hip, knee and ankle.

### 4.3. Subject Assessment

**Figure 13:**
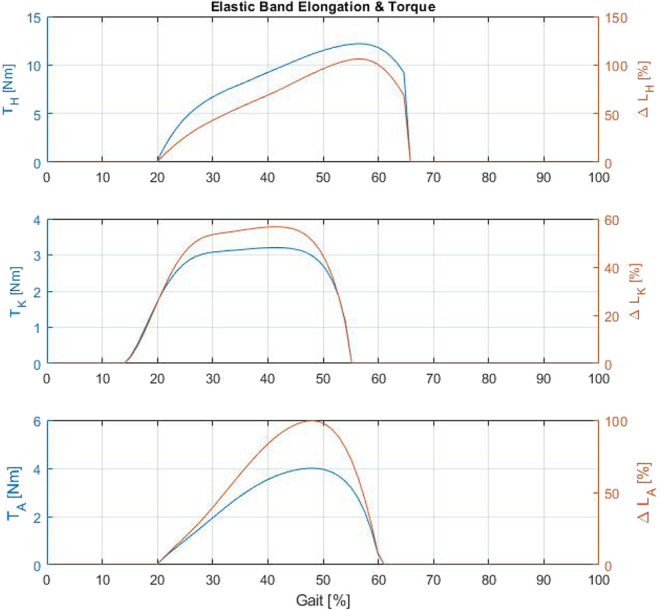
Simulation of elastic band torque and elongation for hip, knee, and ankle.

**Figure 14:**
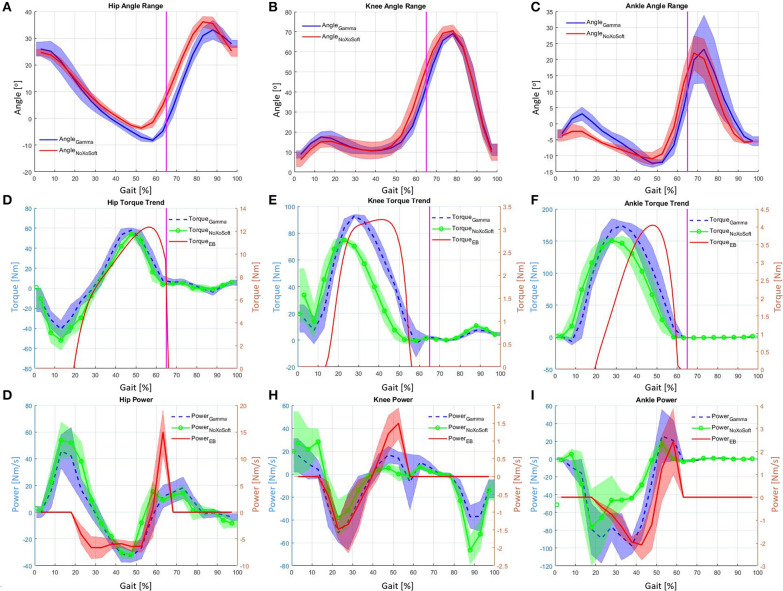
**(A)** Hip angle range wearing XoSoft and not wearing XoSoft. **(B)** Knee angle range wearing XoSoft and not wearing XoSoft. **(C)** Ankle angle range wearing XoSoft and not wearing XoSoft. **(D)** Hip torque wearing XoSoft, not wearing XoSoft and with the EB alone. **(E)** Knee torque wearing XoSoft, not wearing XoSoft and with the EB alone. **(F)** Ankle torque wearing XoSoft, not wearing XoSoft and with the EB alone. **(G)** Hip power wearing XoSoft, not wearing XoSoft and with the EB alone. **(H)** Knee power wearing XoSoft, not wearing XoSoft and with the EB alone. **(I)** Ankle power wearing XoSoft, not wearing XoSoft and with the EB alone.

In this section, the device assessment is addressed. The device was tested on a 10 m long platform with two force plates (BTS P6000, BTS SpA bioengineering, Italy) located about three quarters of way along the course. The tests were completed ten times, during which ground reaction forces on both sides of the body and full body kinematics were measured. Motion capture was recorded using the Xsens system. The AnyBody modeling system (AnyBody Technology A/S, Niels Jernes Vej 10, DK-9220 Aalborg ∅, Denmark) was used to extract and calculate the joint torques from measurement of kinematics and the ground reaction forces. The exoskeleton was configured with six assistive actuators at the hip, knee, and knees on both sides of the body. Three different control sequences were used for frontal hip assistance (hip flexion), rear knee assistance (knee flexion) and rear ankle assistance (ankle plantarflexion). In particular the *QPA*s assisting the hip flexion were triggered from 20 to 65% of the gait cycle. Figure 7 shows that the energy releasing phase is effective until at least 75% of gait cycle. By disengaging the *TBC* at the 65% point, although there is still elastic energy in the system, an instant drop in the assistive torque and elongation occurs (Figure 13). This behavior shows the quality of a *QPA*. It is able to modulate the energy transmitted to the user, by selecting both the instant of activation and release of the actuation during operation. The second pair of *QPA*s assist flexion of right and left knees, with the control triggered at 15% of the gait cycle and clutch disengaged at 55%. Finally, the last pairs of *QPA*s for ankle assistance are triggered between 20 and 65% of gait. Since the ankle angle can exceed the value assumed at the trigger event, the torque transmitted decays to a null value before the end of the control sequence. To demonstrate this, the measured values of ankle shown in Figure 14C (blue trend) shows that the ankle assumes the same angular values at the 20 and 60% of gait cycle. This is also confirmed by the geometrical model, shown in Figure 13, where the *EB* elongation and torque transmission over the ankle joint reach a null value at 60% of the gait cycle. The *EB* elongation and torque generation are based on the model presented in section 3 and validated in section 4. Figure 14 shows the angular displacements, torque and power at the hip, knee and ankle when wearing the *Gamma* prototype and when not wearing it (*NoXoS*), respectively. The torque and power for the *EB* are displayed, showing the net effect of the exoskeleton in assistance mode. When the power is negative the elongation of the *EB* extracts energy from the system (exoskeleton-human) by storing energy, then the net assistance occurs only when the power is positive. The assistive torque causes variations on the measured torque plots. These changes appear as increments or decrements in the total joint torque as the system needs to overcome the *EB* resistance or receives its net assistance.

**Figure 15:**
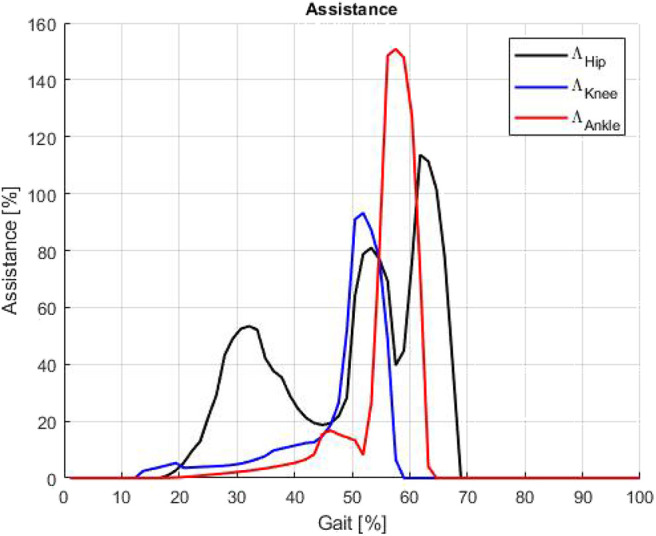
Assistance on hip, knee and ankle expressed as Λ function: the overall average power assistance normalized on the gait are 26.6% for hip, 9.3% for knee, and 12.6% for ankle.

Figure 15 shows the estimate of the exosuit's assistance applying the Equation (10). Which is determined by the ratio of the difference of power at the specific joint with and without the exosuit, with respect to the mechanical power generated at the same joint without having the exoskeleton worn. The power generated wearing the exoskeleton is calculated coupling the measured joint speed and the joint torque estimated applying the geometrical model, which is validated in section 4.1. The diagram clearly shows that the assistance provided may be greater than 100%, because the released net assistive power, in certain gait phases, exceeds the joint power. Thus, the effectiveness of the assistance becomes significant. The power extracted from the system during the *EB* storage phase is less than the characteristic power of the particular gait section. Thus, the storage phase does not affect the overall Λ index, and the user does not feel the extraction of energy from the system. The overall average assistive power measured in terms of the Λ index (shown in Figure 15) for the hip is 26.6%, for the knee 9.3% and for the ankle joint 12.6%. The maximum value of assistive power for the hip is 113.6%, for the knee 93.2% and for the ankle joint 150.8%.

### 4.4. Discussion and Exoskeletons Overview

In this section, we compare the *XoSoft* *Gamma* prototype's main features against the key research and commercial mobile exoskeletons cited in section 1 of this paper and following recent review papers (Li et al., 2015; Yan et al., 2015). Table 7 reports the characteristics of interest of the selected exoskeletons. The main differences is the amount of generated assistance, which also defines the user target. In fact the first two exoskeletons of Table 7, aim to restore the completely loss of mobility for paraplegic patients. Thus, these exoskeletons targets to regain locomotion ability by generating the total amount of joint torque (100% of torque ratio). A second device family aims to provide a partial power to give an aid during walking to people suffering from muscular weakness. An example are elderly people, who need a device able to generate a partial amount of the joint torque. Therefore, if the exoskeleton does not restore total walking faculty the requested assistance reduces within a third of the total joint torque. The natural trend, visible in research, is to move toward a simplification of the structure, which does not necessarily need to be hard but it may become soft. As well, the actuation may be simplified moving from an active to a quasi-passive assistance as demonstrated in the presented device (*XoSoft* *Gamma*). The main differences between a hard structure and a soft structure in terms of comfort and encumbrance are well-underlined in section 1. In general, hard structure are recommended if large amount of torque has to be generated to specific joints. But if that is not the case, the inconveniences related to a heavy and uncomfortable structure arise. Moving from hard to soft a drop of torque ratio is evident, in fact no more than a quarter of the total torque can be generated to the exoskeleton employing soft structure. On the contrary assistive exoskeletons such as the EXPOS and the WWH, can generate a third or half of the total joint torque respectively. Focusing on soft exoskeletons, it is evident that the most common actuation of the selected exoskeletons is the active one. There is only one *QPA* which employing passive elements (e.g., elastic bands) which is able to generate a comparable torque ratio with respect to the active systems but using simpler actuation system.

**Table 7:** Exoskeleton comparison.

**Exoskeleton name**	**Struct. typ**.	**Actuation**	**Joints**	**DOF**	**Bilateral actuation**	**Bidirectional actuation**	**Weight [Kg]**	**Assistive perform. (Torque ratio)**
HAL^+^ (Sankai, 2010)	Hard	AS (P)	Hips, knees, (ankles)	4 (2)	✓	✓	15	100%
Vanderbilt lower-limb orthosis (Farris et al., 2011)	Hard	AS	Hips, knees	4	✓	✓	12	100%
WWH (Nakamura et al., 2005)	Hard	AS	Knees	2	✓	✓	-	50%
EXPOS (Kong and Jeon, 2006)	Hard	AS	Hips, knees	4	✓	✓	3^**^	32%
Power assist wear (Sasaki et al., 2013)	Soft	AS	Knees	2	-	-	3.7	Unknown
Soft Exosuit (Awad et al., 2017)	Soft	AS	Hip	1	-	-	3.2	12%
Soft wearable robotic suit (Jin et al., 2017)	Soft	AS	Hips	2	-	-	2.7	8%
Myosuit (Schmidt et al., 2017)	Soft	AS	Hips, knees	2	-	-	4.6	26%
XoSoft Beta (Di Natali et al., 2019)	Soft	QPA	Hip, knee	2	-	-	2.9	10%
XoSoft Gamma	Soft	QPA	Hips, knees, ankles	6	✓	✓	4	16%*

The acronyms AS means active system, P stances for passive actuation. The value (*) is determined as arithmetical average of the assistance measured at ankle, knee and hip. (^+^) The exoskeleton presents power units for hips and knees, and spring for the ankles joints. The specific exoskeleton marked with (^**^) has a caster walker where motors and transmission are contained.

## 5. Conclusions

This paper presents a novel design and experimental validation of a modular soft exoskeleton for lower limb assistance. Each singular component of the system, such as actuation module, control, and software are evaluated in terms of power consumption, reaction time and force generation. The overall exoskeleton performance is assessed with a healthy user during a walking task. Torque and mechanical power are evaluated, bilaterally, at the three assisted joints (e.g., hip, knee, and ankle). The measured performances in terms of overall assistance, averaged along the gait cycle, are 26.6% for the hip, 9.3% for the knee and 12.6% for the ankle joint. The maximum value of assistive power for the hip is 113.6%, for the knee 93.2% and for the ankle joint 150.8%.

The particular characteristic of this exosuit employing *QPA* to assist joints, as demonstrated in this study, is the ability to accumulate energy in a gait phase (e.g., stance) where the user is generating more power and then released when the joint torque is not very high (e.g., swing). Therefore, the exosuit drains energy from the user to elongate the EB and the user does not feel the extraction of energy from the system. Then, the system releases energy during a gait phase where both the user torque and the assistive torque are comparable. Thus, the released energy affects the user's energy balance more than the stored one. The result, indeed, shows that such system is able to produce a relative power higher than 100% if compared to the actual instantaneous joint torque w/o the exoskeleton (it is shown in Figure 15 after the 50–55% of the gait cycle).

While a statistically relevant study was outside the scope of this current assessment study, these results are very promising and point toward positive findings overall. The device reveals a high potential for energy reduction as well as rehabilitation/motion assistance approaches to address small impairments on specific articulations as has been disclaimed in the following contributions (Graf et al., 2018; Poliero et al., 2018; Sposito et al., 2018; Di Natali et al., 2019). Further studies on patients are being conducted to validate the effectiveness of the exoskeleton. Further improvements to the exoskeleton will aim to develop proprioceptive solutions for sensory based actuators, but also specific soft sensing (Totaro et al., 2017) to measure user's angular joint and enable a more accurate control strategy. Future works will address how XoSoft affects metabolic consumption and muscular activation.

## Data Availability Statement

The datasets generated for this study are available on request to the corresponding author.

## Ethics Statement

The studies involving human participants were reviewed and approved by Comitato Etico Regione Liguria. The patients/participants provided their written informed consent to participate in this study. Written informed consent was obtained from the individual(s) for the publication of any identifiable images or data included in this article.

## Author Contributions

CD contribution on the hardware development, scientific study planning, and conduction of the experimental section and paper drafting. AS, AM, EB, and BH contribution on the hardware development. AD and LO'S contribution on hardware development and proofreading. ER and KS contribution on the control aspects. BM and DC main review aspects. JO scientific review.

## Conflict of Interest

The authors declare that the research was conducted in the absence of any commercial or financial relationships that could be construed as a potential conflict of interest.
